# Exposure Time to a Tuberculosis Index Case as a Marker of Infection in Immigrant Populations

**DOI:** 10.3390/pathogens14020175

**Published:** 2025-02-10

**Authors:** Sofia Godoy, Miquel Alsedà, Ignasi Parrón, Joan-Pau Millet, Joan A. Caylà, Núria Follia, Monica Carol, Angels Orcau, Diana Toledo, Gloria Ferrús, Pere Plans, Irene Barrabeig, Laura Clotet, Angela Domínguez, Jaume March-Llanes, Pere Godoy, on behalf of the Transmission of Tuberculosis in Catalonia (Spain) Working Group

**Affiliations:** 1 Institut de Recerca Biomédica de Lleida (IRBLleida), Universitat de Lleida, 25198 Lleida, Spain; sofiagodoygarcia@gmail.com (S.G.); miquel.alseda@gencat.cat (M.A.); 2 Institut Català de la Salut, 25600 Lleida, Spain; 3 Agència de Salut Pública Catalunya, 08005 Barcelona, Spain; iparron@gencat.cat (I.P.); nfollia@gencat.cat (N.F.); monica.carol@gencat.cat (M.C.); gloria.ferrus@gencat.cat (G.F.); pedro.plans@gencat.cat (P.P.); ibarrabeig@gencat.cat (I.B.); lclotet.mn.ics@gencat.cat (L.C.); 4 Consorcio de Investigación Biomédica en Red de Epidemiología y Salud Pública (CIBERESP), 20029 Madrid, Spain; jmillet@aspb.cat (J.-P.M.); dtoledo@ub.edu (D.T.); angela.dominguez@ub.edu (A.D.); 5 Barcelona Tuberculosis Research Unit Foundation, 08008 Barcelona, Spain; joan.cayla@uitb.cat; 6 Agència de Salut Pública de Barcelona, 08023 Barcelona, Spain; aorcau@aspb.cat; 7 Departament de Medicina, Universitat de Barcelona, 08036 Barcelona, Spain; 8 Departament de Psicologia, Sociologia i Treball Social, Universitat de Lleida, 25001 Lleida, Spain; jaume.march@udl.cat; 9 Hospital Universitari de Santa Maria, 25198 Lleida, Spain

**Keywords:** latent tuberculosis infection, immigrant, tuberculosis, contact tracing, epidemiology, transmission, exposure time

## Abstract

**Background**: Exposure time to a tuberculosis (TB) index case may be a marker of a recent latent tuberculosis infection (LTBI) risk. The aim of this study was to determine the LTBI risk involved in immigrant contact based on exposure time to pulmonary TB index cases. **Methods**: We conducted a 30-month LTBI prevalence study of pulmonary TB immigrant contacts in Catalonia (1 January 2019–30 June 2021). Contacts with LTBI were identified by means of the tuberculin skin test and/or interferon gamma release assay. Variables associated with LTBI in contacts were analysed using adjusted OR (aOR) and 95% confidence interval (CI) values. **Results**: LTBI prevalence was 37.4% (939/2509). Prevalence was higher in men than women (40.6% versus 33.5%; *p* < 0.001), and in all age groups, relative to children <5 years (12.2%; *p* < 0.001)). Prevalence increased with exposure time to the index case; relative to <6 h/week exposure, LTBI risk was greater for both ≥6 h/day (aOR = 2.0; 95% CI: 1.5–2.6) and <6 h/day but ≥6 h/week (aOR = 1.6; 95% CI: 1.1–2.2). **Conclusions**: The LTBI risk in immigrant contacts increases with recent exposure time to the index case, and may suggest recent LTBI in immigrants.

## 1. Introduction

Tuberculosis (TB) elimination is defined as <1 TB case per million people, representing a threshold low enough to ensure that TB will not emerge as a future public health priority [1,2,3]. The objective of eliminating TB is feasible, but even in low-incidence countries, control efforts need to be intensified to achieve elimination in the coming decades [4]. WHO [3] and ECDC [5] guidelines consider the management of latent tuberculosis infection (LTBI) a top priority for TB elimination, particularly for population groups at the highest risk of *Mycobacterium tuberculosis* infection. Priorities for infection control are the contacts of patients with bacteriologically confirmed pulmonary TB (strong recommendation) and immigrants from high-TB-burden areas, prisoners, healthcare workers, homeless people, and recreational drug users (conditional recommendation) [3,6,7,8,9]. Although contact tracing is now protocolized and various guidelines are available [3], recommendations need to be based on empirical data [10,11,12].

Immigrants from high-burden countries are considered a high risk for LTBI, given the likelihood of pre-emigration exposure in the country of origin, exposure on arrival, and exposure during journeys to visit friends and relatives in countries with high transmission levels [13]. Immigrant contacts of recent pulmonary TB cases may show high risks of infection during contact tracing studies; therefore, since exposure time to the index case may be an infection risk marker, it could be useful for the management of LTBI in the immigrant population [14,15,16]. However, that risk should be studied in conjunction with other factors, as it may be modified by contact-related variables, the exposure context and environment, and the characteristics of the index case [13,16,17,18].

In low TB burden regions, such as Catalonia (Spain)—with 8 million inhabitants, 20% of whom are immigrants, and a TB incidence (in 2022) of 13.2 cases per 100,000 inhabitants [19]—contact tracing to detect and treat new LTBI in contacts of pulmonary TB cases may prevent future cases of TB and accelerate the progress of the End TB Strategy [9].

The aim of this study was to determine the LTBI risk of immigrant contact according to exposure time to pulmonary TB index cases.

## 2. Materials and Methods

We carried out an observational study of TB and LTBI prevalence in immigrant contacts of pulmonary TB cases using methods as reported previously [20]. The study population consisted of immigrant contacts of all new pulmonary TB cases recorded by the Catalan epidemiological surveillance network that were identified and in interviewed, as close/casual contacts of index cases in indoor spaces. Inclusion criteria were persons with pulmonary TB, resident in Catalonia, with community and household immigrant contacts who could be traced and investigated. Pulmonary TB cases that met the inclusion criteria were surveyed by public health technicians. The inclusion criteria for contacts were persons born abroad but resident in Catalonia and recently exposed indoors to a pulmonary TB index case registered by an epidemiological surveillance unit.

All recorded contacts were tested with the tuberculin skin test (TST) (n = 1864) and/or the interferon gamma release assay (IGRA) (n = 765). A second test was performed in contacts with a first negative result if no more than 2 months had elapsed since their last contact with the index case. All contacts completed a questionnaire on exposure context and time, cohabitation, smoking habits, and alcohol-related medical risk (>40 g in men and >24 g in women daily or a medical record indicating alcohol abuse). The following health problems were identified (yes/no) from medical records: human immunodeficiency virus (HIV) infection, diabetes, chronic kidney disease, and immunosuppressive disease or treatment.

Immigrant contacts who tested positive for IGRA or TST (≥5 mm) in the first or second test (positive IGRA or >15 mm TST increase) were considered infected and underwent a clinical examination and a posterior–anterior chest X-ray to rule out TB. A possible BCG vaccine booster effect was considered for the 0–4-year age group. Because a TST-boosted BCG immune response can be detected by IGRA, BCG-vaccinated contacts with a positive first or second TST underwent an IGRA to rule out a BCG boosting effect. Following the Catalan Department of Health protocol, the TST was considered negative if the IGRA was negative.

To identify acid-fast bacilli and prepare cultures to rule out the disease, sputum samples were obtained from individuals with lesions suggestive of pulmonary TB. All tests, including polymerase chain reaction (PCR) tests, such as GeneXpert, QuantiFERON^®^-TB Gold Plus, and ELISpot (T-SPOT^®^.TB), were administered by TB clinical units according to Catalan Department of Health protocol.

Data were collected for both index cases and immigrant contacts. Independent variables for the index cases were sputum microbiology and cavitary lesions. For immigrant contacts, the dependent variable was LTBI, and the main independent variables were age, sex, place of exposure (home, school, work, recreational area), and smoking status. Alcohol abuse was also analysed as a marker of exposure in spaces with higher transmission risk. Exposure time to the index case, following the Catalan Department of Health protocol, was recorded in categories as follows: ≥6 h/day; <6 h/day but ≥6 h/week; <6 h/week; and sporadic but intense exposure (limited exposure in high-risk scenarios, such as sharing a car or a small and poorly ventilated space, as rated by the public health technician). Information on exposure time to the index case was collected by public health technician [21].

LTBI prevalence in immigrant contacts was analysed for associations using the odds ratio (OR) and corresponding 95% confidence interval (CI). Statistically significant associations were determined by the chi-square test *p*-value. For the exposure time categories, LTBI risk associated with exposure time to the index case (reference category < 6 h/week) were calculated using OR values as adjusted (aOR) by the unconditional logistic regression method. The variables studied in the multivariate logistic regression model were selected using the backward stepwise method, for a cut-off point of *p* < 0.2. The variables evaluated in the model were exposure time to the index case, age, sex, place of exposure, smoking, alcohol abuse for contacts, and sputum microbiology and cavitary lesions for the index cases.

To study the possible confounding effect of cumulative exposure to the index case, we repeated the statistical analysis with a secondary variable, namely, total exposure hours (calculated as hours from symptom onset to treatment initiation in index cases). Regarding cumulative exposure, LTBI risk was calculated as the aOR (i.e., the OR, adjusted by unconditional logistic regression using the backward stepwise method) using the same variables as for the initial statistical analysis (i.e., cumulative exposure to the index case, age, sex, place of exposure, smoking, alcohol abuse for contacts, and sputum microbiology and cavitary lesions for index cases) (Supplementary Tables S2 and S3).

To avoid the possible booster effect of a second TST in patients with a negative first TST, the data were reanalysed, eliminating individuals with a second TST (positive or negative). The risk associated with LTBI was recalculated as the aOR (and 95% CI), using the same variables from the initial statistical analysis in the regression model (Supplementary Tables S4 and S5).

To detect the effect of exposure time on converters, we restricted the analysis to individuals with a negative first IGRA, and compared individuals with a positive second test with individuals with a negative second test. The effect of exposure time on IGRA conversion was calculated as the aOR (and 95% CI) using a logistic regression model developed using the backward stepwise method with the same variables as used for the initial analysis.

The study was approved by the Ethics Committee of the Arnau Vilanova University Hospital (code: CEIC-2049) and was conducted according to Declaration of Helsinki principles. All subjects included in the study received detailed information on the study aims before inclusion.

## 3. Results

Over the period of study, we recorded 2509 immigrant contacts (55.2% men), with a mean age of 25.8 (standard deviation 18.7) years. Just under half the contacts were aged 18–44 years (18.0% aged 18–29 years and 26.2% aged 30–44 years). The immigrants were generally young, with a low prevalence of comorbidities such as chronic kidney disease (0.3%), cancer (0.4%), HIV infection (0.5%), and diabetes (1.7%) (Table 1). Exposure in the different time categories was as follows: 53.2% (n = 1334) for ≥6 h/day; 17.7% (n = 443) for <6 h/day but ≥6 h/week; 18.4% (n = 462) for <6 h/week; and 6.8% (n = 172) for sporadic but intense exposure (Table 1).

Positive prevalence results for the first TST (38.1%; 709/1864) and IGRA (36.6% 134/765) were similar, as were those for the second TST (17.5%; 134/765) and IGRA (15.1%; 45/297) (Supplementary Table S1).

LTBI prevalence in immigrant contacts, as stratified by the main independent variables (Table 2), was 37.4% (939/2509) overall, was higher in men than in women (40.6% versus 33.5%; *p* < 0.001), and was higher in all age groups compared to children <5 years old (12.2%; *p* < 0.001). Prevalence was also higher in smokers compared to non-smokers (71.5% versus 31.1%; *p* < 0.001), BCG-vaccinated compared to non-BCG-vaccinated persons (43.0% versus 34.9%; *p* < 0.001), and diabetics compared to non-diabetics (66.7% versus 36.9%; *p* < 0.001). Exposure at home (42.3%), in recreational areas (37.2%), and at work (32.4%) led to greater transmission than exposure in schools (23.2%; *p* < 0.001). Regarding exposure, relative to <6 h/week (*p* < 0.001), LTBI risk was greater for ≥6 h/day (OR = 2.5; 95% CI: 1.9–3.1), <6 h/day but ≥6 h/week (OR = 1.5; 95% CI: 1.1–2.0), and for sporadic but intense exposure (OR = 1.8; 95% CI: 1.2–2.6) (Table 2).

Factors associated with LTBI in the logistic regression analysis (Table 3) were as follows: all age groups compared to children <5 years old (*p* < 0.001), male sex (aOR = 1.3; 95% CI: 1.1–1.6); exposure at home (aOR = 3.0; 95% CI: 1.8–4.9) and in recreational areas (aOR = 4.3; 95% CI: 2.1–8.9); and smoking (aOR = 3.4; 95% CI: 2. 6–4.5). Positive sputum smear microscopy (aOR = 1.6; 95% CI: 1.3–1.9) and index case chest X-ray cavitary lesions (aOR = 1.4; 95% CI: 1.1–1.7) were also associated with higher LTBI prevalence, as was increased exposure time to the index case: compared to exposure of <6 h/week, risk was higher for ≥6 h/day (aOR = 2.0; 95% CI: 1.5–2.7), <6 h/day but ≥6 h/week (aOR = 1.6; 95% CI: 1.2–2.3), and sporadic but intense exposure (OR = 1.8; 95% CI: 1.2–2.8) (Table 3).

As for the possible confounding effect of cumulative exposure in LTBI cases, broadly similar results were obtained for the statistical analysis repeated using the secondary variable with four categories based on combinations of exposure days/hours (Supplementary Tables S2 and S3). To assess the booster effect of the second TST and possible infection overestimation, we repeated the analysis eliminating contacts with a second TST. The aOR values estimated for exposure time to the index case and for the other variables in the model were similar to those estimated with the all-data set of patients, with small variations due to the reduced number of persons in this analysis (Supplementary Tables S4 and S5).

The effect of exposure time on conversion was studied in individuals with a negative first IGRA who were retested after 2 months (n = 297). The logistic regression model showed that the risk of conversion increased significantly in the following exposure categories: exposure ≥6 h/day (aOR = 6.8; 95% CI: 1.5–30.9), <6 h/day but ≥6 h/week (aOR = 10.6; 95% CI: 2.2–49.9), and sporadic but intense exposure (OR = 3.3; 95% CI: 0.4–24.1). Note that the confidence intervals overlapped for the different categories, and results were not statistically significant for sporadic but intense exposure category (Table 4).

## 4. Discussion

Our study shows that the LTBI risk in immigrant contacts is associated with exposure time to the index case and that this risk is greater in IGRA converters (contacts with a positive IGRA after a negative IGRA 2 months previously). This finding would indicate that, in order to assess LTBI risk and provide the corresponding treatment, exposure time data need to be systematically collected and assessed in contact tracing studies [22]. Some authors suggest that providing social support to immigrants can help reduce their vulnerability to TB infection, thereby avoiding losses in the LTBI care cascade [23,24].

We found that LTBI risk increased with age and was higher in men and in smokers; transmission risk was also greater for positive sputum smear microscopy and cavitary lesions in the lung parenchyma of index cases. We also found that LTBI prevalence increased as exposure time increased, from 24.2% for <6 h/week, to 33.0% for ≥6 h/week, and to 44.2% for ≥6 h/day, indicating a clear dose–response relationship for those three categories (*p* < 0.001).

Prevalence was also found to be higher (36.6%) for sporadic (not daily) but intense exposure. The fact that this association (aOR = 1.80; 95% CI: 1.16–2.80) was statistically significant in the logistic regression model would indicate that, in addition to time, other factors should be considered, such as the location of exposure, ventilation, and sputum microscopy and cavitary lesions in the index case.

The LTBI association with exposure time to the index case has previously been reported in the literature. Underlining the importance of incorporating exposure time into algorithms to assess LTBI contact risk was the finding, for several TB units in a US study [22], that the LTBI risk increased by 8.2% for every 250 h of cumulative exposure. In our study, prevalence was 38.5% for 201–576 h and 49.0% for >576 h cumulative exposure (Supplementary Table S3). The predictive capacity of exposure time to the index case has also been reported as an LTBI risk in the context of TB outbreaks [14,15]. A previous Catalan study reporting that exposure time to the index case increased the TB risk [20], showed that the risk gradient depending on exposure time went from 0.7% for <6 h/week, to 1.6% for ≥6 h/week and 4.0% for ≥6 h/day, pointing to an evident dose–response relationship for those three categories (*p* < 0.001). Studies on TB outbreaks in different settings [14,15,22,25,26] have highlighted that prolonged exposure to an index case increases the risk of inhaling a higher infectious dose of *M. tuberculosis*, leading to TB infection.

Given that the immigrants in our study were contacts of new and recent cases of pulmonary TB in people residing in Catalonia, exposure time to the index case may suggest that the majority of infections were recent. The increased risk of second IGRA conversion with exposure time to the index case would support this possibility. However, other previous sources of infection, such as travel to the country of origin, should be considered in order to rule out previous infection. This information—collected in accordance with the consensus documents of the Catalan Department of Health [21] and the Tuberculosis Research Unit of Barcelona [27]—may be useful in relation to LTBI treatment prescription, the avoidance of losses in the LTBI care cascade, and the prevention of further TB cases. However, there is no single criterion for studying exposure time; and protocols in other countries and in aircraft have used 8 h of cumulative exposure as a cut-off point for measuring infection risk [5,28].

Another important risk factor is the place of exposure to the index case. In our study, infection risk was increased 3-fold by exposure at home, and 4-fold by exposure in recreational areas. Reichler et al. [22,25,29] documented an OR of 2.6 for exposure at home, while other studies in Spain have reported high infection rates due to exposure to TB index cases in bars [26].

In our study, smoking tripled the risk of LTBI in immigrant contacts—a greater risk than documented in other studies [10,30]. A systematic review (six cross-sectional studies) of persons exposed to second-hand smoke has reported an increased relative risk of LTBI in children and adults of 1.65 and 1.58, respectively [31].

Age was highly relevant regarding the risk of infection. Relative to children <5 years, infection risk was higher in all age groups. The fact that LTBI prevalence was especially high in the 45–64 (57.7%) and the >64 (67.7%) age groups may indicate a higher cumulative risk over time, a greater exposure risk in the country of origin before immigration, or repeated visits to friends and relatives. The fact that women may be less exposed to more contagious TB cases may explain the greater LTBI risk in men [32], which may also be explained by smoking and exposure in recreational areas. According to published studies, immigrants from high TB burden countries systematically present high LTBI prevalence rates [33,34,35,36], due to recent exposure or pre-emigration exposure in the country of origin or after returning from visits to friends and relatives [9,10,37,38,39]. This LTBI risk may be even higher in immigrants from high TB burden countries frequently travelled to for visits to friends and relatives [38,39,40]; in our study, prevalence was especially high in immigrants from Senegal (67.6%; 94/139), Ghana (54.5%; 6/11), Romania (51.8%; 44/85), Morocco (47.8; 204/427), and Gambia (43.2%; 16/37).

Proper LTBI diagnosis is challenging due to the inherent limitations of currently available diagnostic tests. While both the TST and the IGRA measure the adaptive immune response to *M. tuberculosis*, neither can differentiate between recent (<2 years) and non-recent (>2–5 years) infection, between cleared and persistent infection, or between LTBI and active disease [6]. In our study, to rule out TB, contacts with a positive test underwent a clinical examination and a posterior–anterior chest X-ray, while sputum samples were obtained from individuals with lesions suggestive of pulmonary TB to identify acid-fast bacilli, prepare cultures, and implement PCR testing.

IGRA conversion increased with age and exposure time. The lower risk of conversion in children aged under 18 years is likely due to the recommendation to treat probable TB infection in children until a second IGRA is repeated. The increased risk of IGRA conversion with exposure time to TB index cases would support the possibility that many infections were recent, although a dose–response relationship cannot be demonstrated, since the 95% CIs for the different exposure categories overlapped due to the small size of the sample.

Identifying cavitary lesions and positive sputum smear microscopy in index cases and including data on exposure time of contacts to index cases in protocols can be extremely useful to identify LTBI and to inform treatment [10,41,42]. Both factors can modify the role played by contact exposure time. In our study, the LTBI rate was higher when both cavitary lesions and positive sputum smear microscopy were confirmed. In a meta-analysis, Melsew et al. [32] estimated an OR of 2.15 for positive sputum smear microscopy and of 1.90 for cavitary lesions, similar to the rates observed in our study.

Our study has a number of limitations. The risk of LTBI is very high in people aged over 65 years; however, the proportion of immigrant contacts in this age group was very low (1.4%), and so their impact on overall LTBI prevalence was limited. While cumulative exposure time was not considered, similar results were obtained for the analysis that assessed cumulative exposure time from symptom onset to treatment initiation in the index cases (Supplementary Tables S2 and S3). The fact that some TST findings may have been due to BCG vaccination was considered unlikely, as similar TST and IGRA results were obtained for the first and second tests (Supplementary Table S1), while reanalysis of the dataset removing contacts with a second TST showed similar aOR estimates (Supplementary Tables S4 and S5). Immigrant exposure may have occurred in the country of origin, including transmission between humans and ruminants. In our opinion, however, this is unlikely given that all contacts were studied for exposure to a recent TB case unrelated to travel, and an uncovered dose–response relationship was demonstrated between exposure time to the index case and LTBI risk. Nevertheless, we recommend that, for each immigrant contact in contact tracing studies, data on their time in the country and factors related with vulnerability be collected [6]. Although the most likely confounding factors were considered in our multivariate logistic regression analyses, the possibility of residual confounders cannot be ruled out.

Study strengths include rapid and easily collected exposure data in four time categories, inclusion of all contacts (household cohabitants and non-cohabitants), inclusion only of pulmonary TB contacts, retesting of contacts with a recent negative test when no more than 2 months had elapsed since the first test, community health worker participation as cultural mediators and translators, and data collection on the basis of a previously established public health protocol.

Properly assessing LTBI risk in immigrants requires systematic recording of contact exposure time to the index case [22]. In our study, once we controlled for variables such as positive sputum smear microscopy and cavitary lesions in index cases, we found that the LTBI risk associated with exposure of ≥6 h/day and <6 h/day but ≥6 h/week was 2.0 and 1.6 times that of exposure of <6 h/week. The LTBI risk for immigrant smokers and for exposure at home or in recreational areas is known to be high, and our findings suggest that many of these infections originated due to recent exposures.

A cause–effect relationship between exposure time to the index case and TB infection in immigrant contacts is extremely difficult to establish given the variability of transmission over the period of exposure, the difficulties in accurately recording times, and the possibility of exposure of immigrants in or during visits to their country of origin.

Since studies on immigrant contacts of index cases detect significant numbers of LTBI cases, contact tracing and LTBI treatment is key to a TB elimination strategy [43]. Identification of immigrant contacts can be improved by appropriately training public health workers, establishing good rapport with index cases during contact tracing, and implementing specific interventions that take into account the length of time in the host country and the specific needs and vulnerabilities of immigrants. Identification of index cases for contacts, including the exposure time of contacts to index cases, while excluding previous infections due to travel to their country of origin, can be extremely useful in identifying LTBI cases and informing the need for LTBI treatment. In immigrant contact with TB cases, in the case of long exposure times to an index case and a negative IGRA result, the test should be repeated after 8 weeks, since the second test detects a high number of conversions. Additional studies should be performed to establish the cut-off time of exposure to the index case that indicates the level of risk of LTBI in immigrant contacts.

## Figures and Tables

**Table 1:** Characteristics of pulmonary tuberculosis immigrant contacts.

Variable	n (%)
	n = 2509
Age, years	
0–4	551 (22.0)
5–17	354 (14.1)
18–29	453 (18.0)
30–44	658 (26.2)
45–64	428 (17.1)
≥65	31 (1.2)
Unknown	34 (1.4)
Sex	
Male	1383 (55.2)
Female	1123 (44.8)
Exposure time	
≥6 h/day	1334 (53.2)
<6 h/day but ≥6 h/week	443 (17.7)
<6 h/week	462 (18.4)
Sporadic but intense	172 (6.8)
Unknown	98 (3.9)
Smoker	
Yes	393 (15.7)
No/unknown	2116 (84.3)
Place of exposure	
Home	1483 (59.1)
Work	698 (27.8)
Recreational area	94 (3.7)
School	194 (7.7)
Other/unknown	40 (1.6)
BCG vaccination	
Yes	805 (34.2)
No/unknown	1551 (65.8)
HIV	
Yes	12 (0.5)
No/unknown	2497 (99.5)
Diabetes	
Yes	42 (1.7)
No/unknown	2467 (98.3)
Cancer	
Yes	11 (0.4)
No/unknown	2498 (99.6)
Chronic kidney disease	
Yes	7 (0.3)
No/unknown	2502 (99.7)
Index case: positive sputum smear	
Yes	1350 (53.8)
No	1159 (46.2)
Index case: X-ray cavitary lesions	
Yes	1010 (40.3)
No	1499 (59.7)

BCG, bacillus Calmette–Guérin; HIV, human immunodeficiency virus.

**Table 2:** Latent tuberculosis infection (LTBI) risk factors in immigrant contacts.

Variable	LTBI Yes	LTBI No			
	n (%)	n (%)	OR	95% CI	*p* *
Age, years					
0–4	67 (12.2)	484 (87.8)	1.0	Reference	
5–17	132 (37.3)	222 (62.7)	4.3	3.1–6.0	<0.001
18–29	184 (40.6)	269 (59.4)	4.9	3.6–6.8	<0.001
30–44	279 (42.4)	379 (57.6)	5.3	3.9–7.2	<0.001
45–64	247 (57.7)	181 (42.3)	9.8	7.2–13.6	<0.001
>64	21 (67.7)	10 (32.3)	15.0	6.9–34.7	<0.001
Sex					<0.001
Male	562 (40.6)	821 (59.4)	1.4	1.2–1.6	
Female	376 (33.5)	747 (66.5)	1.0	Reference	
Exposure time					
≥6 h/day	590 (44.2)	744 (55.8)	2.5	1.9–3.1	<0.001
<6 h/day but ≥6 h/week	146 (33.0)	297 (67.0)	1.5	1.1–2.1	0.004
<6 h/week	112 (24.2)	350 (75.8)	1.0	Reference	
Sporadic but intense	63 (36.6)	109 (63.4)	1.8	1.2–2.6	0.002
Smoker					<0.001
Yes	281 (71.5)	112 (28.5)	5.6	4.4–7.1	
No/unknown	658 (31.1)	1458 (68.9)	1.0	Reference	
Place of exposure					
Home	627 (42.3)	856 (57.7)	2.4	1.7–3.4	<0.001
Work	226 (32.4)	472 (67.6)	1.6	1.1–2.3	0.017
Recreational area	35 (37.2)	59 (62.8)	2.0	1.1–3.3	<0.012
School	45 (23.2)	149 (76.8)	1.0	Reference	<0.001
BCG vaccination					<0.001
Yes	346 (43.0)	459 (57.0)	1.4	1.2–1.7	
No/unknown	541 (34.9)	1010 (65.1)	1.0	Reference	
HIV					0.760
Yes	5 (41.7)	7 (58.3)	1.2	0.4–3.8	
No/unknown	934 (37.4)	1563 (62.6)	1.0	Reference	
Diabetes					<0.001
Yes	28 (66.7)	14 (33.3)	3.4	1.8–6.5	
No/unknown	911 (36.9)	1556 (63.1)	1.0	Reference	
Cancer					0.015
Yes	8 (72.7)	3 (27.3)	4.9	1.2–17.0	
No/unknown	541 (34.9)	1010 (65.1)	1.0	Reference	
Chronic kidney disease					0.062
Yes	5 (71.4)	2 (28.6)	4.2	0.8–21.7	
No/unknown	934 (37.3)	1568 (62.7)	1.0	Reference	
Index case: positive sputum smear					<0.001
Yes	581 (43.0)	769 (57.0)	1.7	1.4–2.0	
No	358 (30.9)	801 (69.1)	1.0	Reference	
Index case: X-ray cavitary lesions					<0.001
Yes	448 (44.4)	562 (55.6)	1.6	1.4–1.9	
No	491 (32.8)	1008 (67.2)	1.0	Reference	

* *p*-value for the chi-square test. BCG, bacillus Calmette–Guérin; CI, confidence interval; HIV, human immunodeficiency virus; OR, odds ratio.

**Table 3:** Multivariate logistic regression model of latent tuberculosis infection risk in immigrant contacts.

Variable	aOR	95% CI		*p*-Value
Age, years				
0–4	1.00	Reference		
5–17	6.43	4.33	9.55	<0.001
18–29	4.92	3.42	7.09	<0.001
30–44	5.85	4.16	8.23	<0.001
45–64	11.06	7.60	16.10	<0.001
>64	9.57	4.05	22.59	<0.001
Sex				
Male	1.32	1.08	1.61	0.006
Female	1.00	Reference		
Exposure time				
≥6 h/day	2.03	1.50	2.73	<0.001
<6 h/day but ≥6 h/week	1.65	1.18	2.31	0.004
<6 h/week	1.00	Reference		
Sporadic but intense	1.80	1.16	2.80	0.008
Smoker				
Yes	3.42	2.60	4.50	<0.001
No	1.00	Reference		
Place of exposure				
Home	2.96	1.78	4.91	<0.001
Work	1.36	0.82	2.27	0.232
Recreational area	4.31	2.08	8.95	<0.001
School	1.00	-	-	-
BCG vaccination				
Yes	1.13	0.91	1.38	0.248
No/unknown	1.00	Reference		
HIV				
Yes	0.25	0.06	0.92	0.038
No/unknown	1.00	Reference		
Diabetes				
Yes	1.16	0.55	2.43	0.702
No/unknown	1.00	Reference		
Chronic kidney disease				
Yes	2.58	0.37	18.08	0.339
No/unknown	1.00	Reference		
Index case: positive sputum smear				
Yes	1.56	1.26	1.94	<0.001
No	1.00	Reference		
Index case: X-ray cavitary lesions				
Yes	1.40	1.13	1.74	0.002
No	1.00	Reference		

aOR, odds ratio adjusted to the variables in the table; BCG, bacillus Calmette–Guérin; CI, confidence interval; HIV, human immunodeficiency virus.

**Table 4:** Multivariate logistic regression model of interferon gamma release assay (IGRA) conversion in immigrant contacts of index tuberculosis cases.

Variable	Total	IGRA Positive			
		n (%)	aOR	95% CI	*p*-Value
Age, years					
0–17	161	10 (6.2)	1.00	Reference	
18–29	43	8 (18.6)	6.79	1.72–26.78	0.006
30–44	56	13 (23.2)	10.57	3.07–36.35	<0.001
45–64	37	14 (37.8)	23.51	6.37–82.08	<0.001
Sex					0.960
Male	156	26 (16.7)	0.98	0.44–2.20	
Female	141	19 (13.5)	1.00	Reference	
Exposure time					
≥6 h/day	184	24 (13.0)	6.79	1.49–30.90	0.013
<6 h/day but ≥6 h/week	65	14 (21.5)	10.61	2.25–49.94	0.003
<6 h/week	32	4 (12.5)	1.00	Reference	-
Sporadic but intense	16	3 (18.7)	3.30	0.45–24.11	0.239
Smoker					0.133
Yes	31	14 (45.2)	2.36	0.77–7.25	
No/unknown	266	31 (11.6)	1.00	Reference	
BCG vaccination					0.004
Yes	172	22 (12.8)	0.26	0.10–0.66	
No/unknown	113	20 (17.7)	1.00	Reference	
Index case: positive sputum smear					0.203
Yes	146	28 (19.2)	1.87	0.73–7.86	
No	151	17 (11.3)	1.00	Reference	
Index case: X-ray cavitary lesions					0.002
Yes	94	28 (29.8)	3.99	1.64–9.66	
No	203	17 (8.4)	1.00	Reference	

aOR, odds ratio adjusted to the variables in the table; CI, confidence interval. BCG, bacillus Calmette–Guérin.

## Data Availability

The dataset is available from the corresponding author upon reasonable request.

## Supplementary Materials

The following supporting information can be downloaded at https://www.mdpi.com/article/10.3390/pathogens14020175/s1, Table S1: Positive test results for the first and second TST and IGRA in immigrant contacts of tuberculosis index cases; Table S2: LTBI risk in immigrant contacts by accumulated exposure to tuberculosis index cases; Table S3: Multivariate logistic regression model of LTBI risk in immigrant contacts; Table S4: LTBI risk in immigrant contacts by accumulated exposure to tuberculosis index cases, excluding individuals with a second TST; Table S5: Multivariate logistic regression model of LTBI risk in immigrant contacts, excluding individuals with a second TST.
