# Supplementary material for: Exposure Time to a Tuberculosis Index Case as a Marker of Infection in Immigrant Populations
Source: Pathogens. 2025 Feb 10;14(2):175. doi: 10.3390/pathogens14020175 (PMC11858108; doi:10.3390/pathogens14020175)
Supplement: Supplementary file 1 [file pathogens-14-00175-s001.zip › pathogens-3379006-supplementary.pdf]

Supplementary Table S1. Positive test results for the first and second TST and IGRA in immigrant contacts of tuberculosis index cases

Test	Positive result n/N	(%)
First TST	709/1864	(38.01)
Second TST	134/765	(17.5%)
First IGRA	280/764	(36.6)
Second IGRA	45/297	(15.1)

IGRA, interferon gamma release assay; TST, tuberculin skin test.

Supplementary Table S2. LTBI risk in immigrant contacts by accumulated exposure to tuberculosis index cases

Variable	LTBI Yes, N (%)	LTBI No, N (%)	OR	95% CI	p*
Age, years					
0 – 4	67 (12.2)	484 (87.8)	1.0	Reference	
5 – 17	132 (37.3)	222 (62.7)	4.3	3.1-6.0	<0.001
18 – 29	184 (40.6)	269 (59.4)	4.9	3.6-6.8	<0.001
30 – 44	279 (42.4)	379 (57.6)	5.3	3.9-7.2	<0.001
45 – 64	247 (57.7)	181 (42.3)	9.8	7.2-13.6	<0.001
>64	21 (67.7)	10 (32.3)	15.0	6.9-34.7	<0.001
Sex					
Male	562 (40.6)	821 (59.4)	1.4	1.2-1.6	<0.001
Female	376 (33.5)	747 (66.5)	1.0	Reference	
Accumulated exposure, hours					
0-42	61 28.6)	152 (71.4)	1.0	Reference	
43-200	163 (34.5)	309 (65.5)	1.3	0.9-1.9	0.064
201-576	161 (38.3)	260 (61.8)	1.5	1.1-2.2	0.008
>576	239 (49.0)	249 (51.0)	2.4	1.7-3.4	<0.001
Smoker					
Yes	281 (71.5)	112 (28.5)	5.6	4.4–7.1	<0.001
No/unknown	658 (31.1)	1458 (68.9)	1.0	Reference	
Place of exposure					
Home	627 (42.3)	856 (57.7)	2.4	1.7-3.4	<0.001
Work	226 (32.4)	472 (67.6)	1.6	1.1-2.3	0.017
Recreational area	35 (37.2)	59 (62.8)	2.0	1.1-3.3	<0.012
School	45 (23.2)	149 (76.8)	1.0	Reference	<0.001
Index case: positive sputum smear					
Yes	581 (43.0)	769 (57.0)	1.7	1.4-2.0	<0.001
No	358 (30.9)	801 (69.1)	1.0	Reference	
Index case: Rx cavitary lesions					
Yes	448 (44.4)	562 (55.6)	1.6	1.4-1.9	<0.001
No	491 (32.8)	1008 (67.2)	1.0	Reference	

*p-value for the chi-square test.

CI, confidence interval; LTBI, latent tuberculosis infection; OR, odds ratio.

Supplementary Table S3. Multivariate logistic regression model of LTBI risk in immigrant contacts

Variable	aOR	95% CI		p
Age, years				
0 – 4	1.00	-	-	-
5 – 17	9.30	5.90	14.69	<0.001
18 – 29	5.20	3.37	8.02	<0.001
30 – 44	7,.63	5.17	11.25	<0.001
45 – 64	12.35	7.99	19.08	<0.001
≥64	4.98	1.80	13.78	<0.001
Sex				
Male	1.36	1.10	1.74	0. 016
Female	1.00	-	-	-
Accumulated exposure, hours				
0-42	1.00	-	-	-
43-200	1.46	0.95	2.23	0.084
201-576	1.77	1.14	2.76	0.011
>576	2.44	1.57	3.78	0.001
Smoker				
Yes	4.11	2.98	5.67	<0.001
No/unknown	1.00	-	-	-
Place of exposure				
Home	2.51	1.40	4.52	0.002
Work	0.78	0.42	1.44	0.426
Recreational area	2.82	1.23	6.49	0.014
School	1.00	-	-	-
Index case: positive sputum smear				
Yes	1. 19	0.90	1.58	0.223
No	1.00	-	-	-
Index case: Rx cavitary lesions				
Yes	2.02	1.51	2.70	<0.001
No	1.00	-	-	-

aOR, adjusted odds ratio; LTBI, latent tuberculosis infection.

Supplementary Table S4. LTBI risk in immigrant contacts by accumulated exposure to tuberculosis index cases, excluding individuals with a second TST

Variable	LTBI				
	Yes, N (%)	No, N (%)	OR	95% CI	p*
Age, years					
0 – 4	59 (18.1)	266 (81.8)	1.0	Reference	
5 – 17	112 (52.1)	103 (47.9)	4.9	3.3-7.2	<0.001
18 – 29	163 (48.4)	174 (51.6)	4.2	3.0-6.0	<0.001
30 – 44	248 (51.1)	237 (48.9)	4.7	3.4-6.6	<0.001
45 – 64	219 (42.3)	119 (35.2)	8.2	5.8-11.9	<0.001
>64	21 (77.8)	6 (22.2)	15.6	6.2-44.0	<0.001
Sex					0.011
Male	492(50.3)	486 (49.7)	1.3	1.1-1.5	
Female	338 (44.2	427 (55.8)	1.0	Reference	
Exposure time					
≥6 hours/day	532 (52.9)	474 (47.1)	1.9	1.4-2.5	<0.001
<6 hours/day but ≥6 hours/week	117 (42.1)	161(57.9)	1.2	0.9-1.7	0.228
<6 hours/week	105 (37.1)	178 (62.9)	1.0	Reference	
Sporadic but intense	58 (44.6)	72 (55.4)	1.4	0.9-2.1	0.147
Smoker					
Yes	255 (75.0)	85 (25.0)	4.3	3.3–5.6	<0.001
No/unknown	576 (41.0)	828 (59.0)	1.0	Reference	
Place of exposure					
Home	585 (51.4)	552 (48.5)	3.7	2.4-6.0	<0.001
Work	184 (44.3)	231 (55.7)	2.8	1.7-4.7	<0.001
Recreational area	33 (48.5)	35 (51.5)	3.3	1.7-6.4	<0.001
School	25 (22.1)	88 (77.8)	1.0	Reference	
Vaccination BCG					
Yes	309 (47.8)	338 (52.2)	1.0	0.8–1.2	0.880
No/unknown	479 (47.4)	532 (52.6)	1.0	Reference	
VIH					
Yes	5 (45.4)	6 (54.5)	0.9	0.3–3.0	0.883
No/unknown	826 (47.7)	907 (52.3)	1.0	Reference	
Diabetes					
Yes	27 (71.1)	11 (28.9)	2.7	1.4–5.6	0.003
No/unknown	804 (47.1)	902 (52.9)	1.0	Reference	
Cancer					
Yes	8 (72.7)	3 (27.3)	2.9	0.8–11.1	0.094
No/unknown	823 (47.5)	910 (52.5)	1.0	Reference	
Chronic kidney disease					
Yes	5 (100.0)	0 (0.0)	-	-	0.019
No/unknown	826 (47.5)	913 (52.5)	1.0	Reference	
Index case: positive sputum smear					
Yes	532 (54.1)	452 (45.9)	1.8	1.5-2.2	<0.001
No	299 (39.3)	461 (60.6)	1.0	Reference	
Index case: Rx cavitary lesions					
Yes	404 (58.2)	290 (41.8)	1.6		<0.001

No	427 (40.7)	623 (59.3)	2.0	1.7-2.5<br>Reference	

*p-value for the chi-square test.

CI, confidence interval; LTBI, latent tuberculosis infection; OR, odds ratio.

Supplementary Table S5. Multivariate logistic regression model of LTBI risk in immigrant contacts, excluding individuals with a second TST.

Variable	aOR	95% CI		p
Age, years				
0 – 4	1.00	-	-	-
5 – 17	4.32	2.82	6.60	<0.001
18 – 29	3.18	2.15	4.70	<0.001
30 – 44	4.08	2.83	5.88	<0.001
45 – 64	6.74	4.53	10.00	<0.001
≥64	13.79	4.47	42.50	<0.001
Sex				
Male	1.10	0.88	1.37	0. 382
Female	1.00	-	-	-
Exposure time				
≥6 hours/day	1.00	-	-	-
<6 hours/day but ≥6 hours/week	2.21	1.64	2.98	<0.001
<6 hours/week	1.47	1.00	2.15	0.049
Sporadic but intense	1.43	0.88	2.32	0.149
Smoker				
Yes	2.95	2.19	3.99	<0.001
No/unknown	1.00	-	-	-
Vaccination BCG				
Yes	1.01	0.81	1.27	0.895
No/unknown	1.00	-	-	
VIH				0.044
Yes	0.24	0.06	0.96	
No/unknown	1.00	-	-	
Diabetes				
Yes	1.17	0.53	2.58	0.700
No/unknown	1.00	-	-	
Index case: positive sputum smear				
Yes	1. 46	1.15	1.84	0.002
No	1.00	-	-	-
Index case: Rx cavitary lesions				
Yes	1.68	1.32	2.13	<0.001
No	1.00	-	-	-

aOR, adjusted odds ratio; LTBI, latent tuberculosis infection.
